# Pandemic preparedness systems and diverging COVID-19 responses within similar public health regimes: a comparative study of expert perceptions of pandemic response in Denmark, Norway, and Sweden

**DOI:** 10.1186/s12992-022-00799-4

**Published:** 2022-01-21

**Authors:** Jakob Laage-Thomsen, Søren Lund Frandsen

**Affiliations:** Department of Organization, Copenhagen Business School, Frederiksberg, Denmark

**Keywords:** Pandemic preparedness, COVID-19, Expertise, Comparative analysis, Policy studies

## Abstract

**Background:**

National responses to the COVID-19 pandemic depend on national preparedness systems that must be understood as components of global public health emergency preparedness systems, governed and coordinated through the World Health Organization’s 2005 International Health Regulations. The pandemic has raised the question of why countries belonging to similar public health regimes, coordinated through the same global system, responded differently to the same threat. Comparing the responses of Denmark, Sweden and Norway, countries with similar public health regimes, the paper investigates to what degree national differences in COVID-19 policy response reflect significant differences in the policy preferences of national expert groups.

**Results:**

We employ a structured case comparison of Denmark, Norway, and Sweden to analyze their’ politico-administrative pandemic preparedness systems and policy responses during the first wave of the COVID-19 pandemic. We use the results of an interdisciplinary expert survey completed in 2020 to analyze expert perceptions in two ways. First, we analyze expert perceptions of COVID-19 responses while controlling for national COVID-19 trajectories and experts’ characteristics. Second, we analyze the distribution and effect of dominant global expert-held ideas across countries, showing the importance of dominant ideas for experts’ perceptions and preferences for COVID-19 response.

**Conclusion:**

The study finds no evidence indicating that COVID-19 policy variation between the most similar cases of Denmark, Norway, and Sweden are the result of differences in the policy preferences of national expert groups. Instead, our study highlights the importance of other factors than cross-national expert dissensus for explaining variation in pandemic response such as the politico-administrative organization of pandemic preparedness systems. Further, we find that expert support for dominant ideas such as a ‘focused protection strategy’ is associated with consistent policy preferences across locational, disciplinary, and geographic affiliations. Recognition of the latter should be a part of future discussions about how global ideas of pandemic preparedness are diffused transnationally and embedded in national politico-administrative systems.

**Supplementary Information:**

The online version contains supplementary material available at 10.1186/s12992-022-00799-4.

## Background

Strengthening pandemic preparedness systems is central to reduce the impact of pandemics on vital societal functions [28]. In many countries the COVID-19 pandemic has unsettled belief in the ability of existing preparedness principles and institutions to properly mitigate global health emergencies. Recent studies have highlighted significant variations in how governments have understood and acted upon the pandemic to minimize its consequences, resulting in large national differences in mortality rates and economic repercussions [10]. Importantly, such policy divergences have crystallized despite the World Health Organization’s (WHO) efforts to strengthen and harmonize national pandemic preparedness since the late 1990s [55]. The inadequacy of prior assessments of pandemic preparedness in predicting COVID-19 efficacy has already been documented, such as in the case of the 2019 Global Health Security Index (GHSI), which ranked the US and the UK number one and second in the world in relation to health security capabilities prior to the pandemic [33]. The predictions of rankings such as the GHSI have since received widespread criticism as a result of the US and the UK’s initial COVID-19 responses [18], displaying, in turn, a number of issues in the organization of established pandemic preparedness systems [19].

A starting point for exploring these issues is to compare how globally configured pandemic preparedness systems governed and coordinated through the WHO’s 2005 International Health Regulations were activated in countries with similar public health regimes [27, 35]. Recent studies have shown how countries with the same public health regime responded differently to the COVID-19 pandemic during its first wave despite similarities in health expenditures and health infrastructure [7, 10]. Such within regime variation has fertilized the ground for discussions about what types of public health regimes (democratic or authoritarian), and formal political institutions (federalism or presidentialism) best determine COVID-19 response and performance [27, 37]. While these determinants are highly useful for discussing the macro-politics of global health emergency response, they, however, provide little guidance for understanding COVID-19 policy divergence between countries belonging to the same public health regime.

A useful point of departure for understanding such divergence among most similar countries are the Nordic welfare states’ different responses to the COVID-19 pandemic. Denmark, Norway, and Sweden engaged differently with the pandemic during its first wave despite sharing Social Democratic and Scandinavian welfare state models, health care organization, geography, corporatist traditions, levels of institutional trust, and high rankings in the GHSI (Denmark 8; Norway 16; Sweden 7) [11, 48]. Differences in the use of lockdown measures, face masks, testing, and COVID-19 response strategies raises the question of how countries belonging to similar public health regimes could respond so differently to the same biological threat.

In studying the policy process, policy studies have long recognized expert-based information as one of the most important factors for explaining policy outcomes [64]. More than just information itself, research in the tradition of Policy Advisory Systems (PAS) have underlined the importance of understanding the sources of policy advice, both within and outside bureaucracies, as always operating within particular ‘systems’ of policy advisors that vary between jurisdictions and change over time [14, 31]. In this framework, policy influence has been conceptualized as a function of location within the system and the type of advice being offered [15]. Recent theoretical developments in the ‘second wave’ of PAS has transposed this framework to better grasp the ‘context’ and ‘content’ of policy advice [16]. Particularly, issues of ‘context’ have led to an incorporation of key insights from the policy subsystems literature, shifting, in turn, analytical emphasis to the policy subsystem context in which advisory systems are situated. The basic idea is that the structure of policy subsystems, defined as the decision-making networks structured around policy issues, affect the influence and access of various sources of policy advice in important ways (ibid., [64]).

The importance of policy advice is particularly salient in the policy subsystem of pandemic preparedness, where research on public health security have long emphasized the centrality of experts in the design and implementation of pandemic preparedness systems [39]. Here, effective policy making in response to complex health crises is highly dependent on expert-based advice and information [53]. Experts occupy influential positions within the subsystem, such as in public health agencies and advisory committees where they are involved in framing biological threats and defining policy measures to respond to them [46, 47, 54]. This makes them important gatekeepers for understanding variation between the otherwise esoteric, technical, and siloed national policy subsystems activated during the COVID-19 pandemic [39, 52].

Studies of responses to prior pandemics such as H1N1 in 2009 have highlighted the importance of expert norms for understanding different national responses to health emergencies [3, 4, 52]. Here, expert advisors enacted ideas about the pandemic threat, settled during preparedness planning and from past experiences, which shaped policymaking. Despite public health policy in the Nordics traditionally being described as what might be called a ‘unitary subsystem’, with centralized authority in public health agencies, dominant policy images coordinated through the WHO and high intra-coalition belief compatibility [64], responses to the first wave of the pandemic saw conflicting expert visions and advice for the most appropriate national response strategy to the COVID-19 pandemic. In Sweden, in particular, these disagreements spilled out onto the pages of The Lancet [12, 25] and in public signature collections [17], pitting experts sympathetic or critical to the policies of the Swedish health authorities against each other. Globally, similar diverging positions of policy advice further crystallized with the publications and signature collections of the ‘Great Barrington Declaration’ and the ‘John Snow Memorandum’, which promoted opposite strategies to the application of non-pharmaceutical interventions like societal-wide lockdowns [9]. The existence of conflicting COVID-19 policy advice, in both the general public and in specialized research outlets, raises the question of the extent to which pandemic response divergences between countries belonging to the same public health regime are the result of differences in the ideas, attitudes, and policy preferences of national expert groups.

In light of these developments, this paper asks how countries within the same public health regime diverged in policy response to the COVID-19 pandemic and, given the central role of experts and expert-based information as a potential source of policy divergence, to what degree this divergence reflects important differences in the policy preferences within and between national expert groups?

To answer the research question, the paper employs a structured case comparison of the three Nordic countries Denmark, Norway, and Sweden, drawing on a mixed methods design. After presenting the methods in more detail, the first part of the paper compares the three countries’ politico-administrative pandemic preparedness systems and their responses to the first wave of the COVID-19 pandemic. The second part of the paper investigates to what degree these responses had support among and reflected dominant ideas held by national experts varying in disciplinary backgrounds and locations in the policy advisory system of pandemic preparedness. To do so, the paper presents and analyzes the results of an interdisciplinary and cross-country expert survey completed by 232 respondents from November–December 2020. It does this by, first, analyzing expert perceptions of national responses to the pandemic using a multidimensional response measure. Second, it applies an ordinal regression model to analyze to what degree experts’ attitudes to their respective governments efforts to save lives of citizens are associated with ideational commitment among experts to ‘focused protection’ or ‘community protection’ while controlling for the locational characteristics of experts in the policy advisory system. The analysis is followed by a discussion of the relationship between dominant ideas in national expert groups and perceptions of COVID-19 responses.

The paper makes a substantive contribution to the existing literature on public health security in general, and the COVID-19 pandemic in particular, by examining and comparing attitudes and policy preferences for national pandemic responses among national experts belonging to similar public health regimes. When controlling for locational positions in politico-administrative systems, disciplinary affiliations, as well as demographic and sampling factors, we find that experts present in countries pursuing a suppression strategy dependent on non-pharmaceutical interventions such as societal-wide lockdowns like Denmark and Norway systematically perceived their country’s COVID-19 response more favorably in terms of saving lives compared to experts from a country pursuing a mitigation strategy without the use of lockdown measures like Sweden. In light of our comparison of the three countries pandemic preparedness systems and the result of our analysis of expert attitudes to pandemic responses, we suggest that policy divergence is more related to politico-administrative factors than policy preferences among national expert communities.

Pandemic preparedness systems are defined as the set of institutions established to prepare and respond to infectious disease emergencies to mitigate their impact on systems critical to the functioning of society [13]. ‘Preparedness’ itself captures the idea that societies cannot prevent catastrophic events, like pandemics, but can prepare for them to mitigate their consequences. This requires the identification of vulnerabilities through the enactment of imagined scenarios, the development of plans to handle potential emergencies, and investments in response capacities and equipment. Despite the fact that preparedness is often treated ‘in potentia’, the circumscribed systems are part of an emergency management cycle of anticipation, response and recovery that was activated during the initial response to the spread of the SARS-CoV-2 virus [50]. The ‘policy issue’ of pandemic preparedness thus also demarcates the policy subsystem from which experts were sampled.

Since the late 1990s, the WHO has been instrumental in diffusing and standardizing the concept of pandemic preparedness across the world (Kamradt-Scott and McInnes [34]). The WHO has published several reports advocating pandemic influenza preparedness and has pushed its members to develop pandemic preparedness systems. Denmark, Norway, and Sweden established pandemic preparedness systems in the early 2000s, heavily influenced by the work of the WHO. As the WHO declared COVID-19 a “Public Health Emergency of International Concern” on the 31st of January 2020, these systems were set in action and later called into question when the three countries diverged on COVID-19 policy.

## Methods

This study employs a structured comparative most similar case design to explain variation and similarity in pandemic preparedness systems and first wave COVID-19 responses in three similar countries, Denmark, Norway, and Sweden, with different COVID-19 response policies [24]. The design consists of two parts, combined in a mixed methods research strategy, to avoid the risks of selective evidence sometimes associated with qualitative research, while yielding nuanced accounts that supplement statistical associations [59]. The first part systematically compares pandemic preparedness systems and COVID-19 response strategies in the three countries. The second part subjects an expert survey on perceptions of COVID-19 response to quantitative analysis.

### Structured case comparison

It is particularly useful to compare Denmark, Norway, and Sweden’s responses to the COVID-19 pandemic because the three Nordic countries share many political, public health and social characteristics [52]. The countries are similarly located in Northern Europe, making their populations exposure to fast-spreading infectious diseases such as COVID-19 similar. They share linguistic, cultural, and political characteristics. They are all Social Democratic welfare states with universal health care [20] and wealthy coordinated market economies [30]. The three countries are most often grouped together in ‘welfare state’ and ‘actor-constellation-based’ typologies of public health regimes, although one recent ‘institutional design’ typology has placed Sweden in category with Finland, Portugal, and Spain [51, 65]. This makes them similarly capable of managing infectious disease cases while also giving them similar financial capacity to install testing capacities, technologies, and preparedness investments [52]. In light of these similarities, a comparison of the three countries control a variety of factors that can otherwise plausibly explain variation in COVID-19 response policy. It is thus useful for analyzing to what extend COVID-19 policy divergence between most-similar countries are the result of diverging policy preferences and ideas among national expert groups.

The structured case comparison of the three countries is limited to four important dimensions central to the politico-administrative organization of national pandemic preparedness systems: (1) the autonomy of health expertise in the state; (2) health care system centralization; (3) lowest administrative level of pandemic preparedness system; and (4) COVID-19 response strategy. The first dimension refers to the autonomy of health experts in preparing for and responding to health emergencies in the state, in other words the power and responsibilities of public health experts and state agencies to define threats, risk scenarios and steer policy as they see fit vis-a-vis elected politicians [8]. It is thus related to whether political leaders are able to intervene in the daily operations of public health agencies during pandemics as well as the principles that guide expert-politician interaction. The second dimension refers to the degrees of centralization or decentralization in national health care systems, that is, the policy power given to central or less central authorities within the system [42]. The third dimension refers to the lowest administrative level at which public health interventions to prepare and respond to infectious disease emergencies are implemented. This dimension is important for understanding the actors present in the policy advisory system around the issue of pandemic preparedness. The final dimension refers to the COVID-19 response strategies employed in the three countries during the first wave of the pandemic. This is a relevant dimension to include as it enables us to distinguish different national response strategies from each other. We draw on Ferguson et al. [23] to distinguish between three COVID-19 strategies: containment, suppression, and mitigation. Containment strategies focus on isolating and containing the virus to stop it from entering into the country in the first place through border closures, travel restrictions and quarantine measures. Suppression and mitigation are two response strategies for handling the pandemic once the virus has already spread to significant parts of the population. Mitigation refers to a strategy whereby transmission is reduced but the effective reproduction number (R_t_) remains above 1 to build up herd immunity in the majority of the population through natural infection while isolating individuals at risk. This strategy does not adopt strong non-pharmaceutical measures like lockdowns. While mitigation can have more encompassing meanings in the literature, this use of the concept is specifically tied to Ferguson et al. [23] classification of COVID-19 strategies. Suppression, oppositely, refers to a strategy whereby transmission is reduced to keep R_t_ below 1 through non-pharmaceutical interventions like societal wide lockdowns until population-wide vaccination is possible.

The dimensions are analytically driven. The politico-administrative organization of pandemic preparedness systems is important for limiting the space in which authorities are able to make decisions [27]. Meanwhile, in combination with the COVID-19 strategy, it also constitutes the basis on which expert perceptions are formed, and to which they must be related analytically. The comparison relies on official documents like pandemic preparedness plans and commission reports, national and international peer-reviewed articles and quantitative policy indicators.

### Expert survey

We investigated the perceptions of experts on COVID-19 response and pandemic preparedness through a cross-national expert survey of 232 experts with disciplinary backgrounds in medicine, public health, epidemiology, virology, and economics. Experts were sampled from a database of all national public experts constructed between January–September 2020 (see Appendix A). This sampling strategy was chosen for two reasons. First, given the number of disciplines accounting for and relevant to pandemic preparedness [43], we sought to create a maximum variation of COVID-19 relevant expertise. For this reason, sampling strategies based on institutional affiliation or snowballing were disfavored due to the risk that they might reinforce disciplinary divides. News media are stable empirical sources for comparative analysis across countries and have shown to be an important site for expert statements, contestation, and discussion [1]. Second, public presence indicates expertise relevant to understanding and handling the pandemic, which pre-analysis confirmed. Public visibility is a key definitional element of expertise [22], and to our knowledge, this study is the first to systematically map and display the different forms of expertise related to pandemic preparedness in the Nordics.

Experts were selected based on two criteria: a formal association with a national university, hospital, research institution or public agency, and work within aforementioned disciplines. These disciplinary backgrounds were selected as they matched the economic and health dimensions of preparedness, which the survey sought to address. To control for selection biases associated with public media presence, an endogenous measure in the form of “presence on institutional expert lists” was incorporated into the design. Many universities and hospitals have published lists of institutionally identified experts in light of the pandemic. We collected publicly available lists from institutions represented by a minimum of two experts in the initial dataset. This search yielded a control group of 208 experts, of which more than half were already in the dataset based on news media presence.

Based on this population sampling strategy, we identified 982 experts that were sent an online survey via email, open from November 17 to December 20. We collected 232 complete responses, corresponding to a response rate of 24%. We performed a nonresponse bias analysis (see Appendix A) which did not reveal any major underrepresented expert subgroups, although women and individuals with positions in hospitals or management functions had lower response rates. We return to the study’s main limitations in the discussion. The survey was pre-tested with experts in all countries and divided into five sections. The first section collected expert attributional data. The second collected data on the experts’ advisory activities prior to and during the pandemic. The third section asked respondents to evaluate their national preparedness systems in relation to eight dimensions before COVID-19 and their government’s reaction to the pandemic, while the fourth section asked how pandemic preparedness might be improved in the future. The final section presented the respondents with a selection of “pandemic puzzles” related to known points of expert disagreement and policy suggestions.

### Quantitative analysis

We conducted statistical analysis of the select variables of the expert survey. The descriptive statistics of the survey population can be found in Table 1. The analysis begins with a descriptive analysis of experts’ perceptions of pandemic response using a multi-dimensional measure of pandemic response along eight dimensions: saving lives of citizens, securing integrity of critical infrastructure, prevent economic recession, prevent socio-economic inequality, secure state finances, ensure citizens’ mental health and wellbeing, protect vulnerable groups, and maintain democratic accountability. We asked experts “In the period from the detection of the first COVID-19 case in your country and until the beginning of October 2020, how would you assess the performance of your country’s COVID-19 response activities? Respondents were able to answer on an ordinal scale from ‘underreacted’ (government reacted with less than appropriate resources), to ‘neither or’, and ‘overreacted’ (government reacted with more than appropriate resources). These answer categories were chosen to allow experts to answer using both ends of the reaction spectrum (underreaction/neither-or/overreaction). Respondents were also able to answer “don’t know/not relevant”. We report the results in Fig. 2 in the result section.

**Table 1:** Descriptive statistics of survey population

Saving lives (main dependent variable)	
Underreaction	64 (27%)
Neither underreaction nor overreaction	129 (56%)
Overreaction	39 (17%)
**Dominant Ideas (main independent variable)**	
***Supporting focused protection, N Experts (%)***	
No (baseline)	175 (75%)
Yes	57 (25%)
**National Factors**	
***Country, N Experts (%)***	
*Norway (baseline)*	53 (23%)
*Denmark*	88 (38%)
*Sweden*	91 (39%)
**Locational Factors**	
***National Public health institution, N Experts (%)***	
No (baseline)	183 (79%)
Yes	49 (21%)
***Seniority, N Experts (%)***	
Senior (Full professorship or Chief Physician) (baseline)	132 (57%)
Management	22 (9%)
Other (less than senior or management, eg. associate/assistant professor, MD)	78 (34%)
***Advised National Government, N Experts (%)***	
No (baseline)	113 (49%)
Yes	119 (51%)
**Disciplinary factors, N Experts (%)**	
Medicine (baseline)	109 (47%)
Public Health	57 (25%)
Economics	36 (15%)
Lab	30 (13%)
**Demographic and geographic factors**	
***Gender, N Experts (%)***	
Male (baseline)	169 (73%)
Female	63 (27%)
***Age, Year, mean ± SD (range)***	
Age	57 ± 11 (46–68)
***Metropolitan regions, N Experts (%)***	
No (baseline)	69 (30%)
Yes	163 (70%)
**Sampling factors**	
***On expert list, N Experts (%)***	
No (baseline)	173 (75%)
Yes	59 (25%)

We then use experts’ perceptions of their national government’s response to the COVID-19 outbreak in relation to the dimension ‘saving lives of citizens’ as the dependent variable in an ordinal regression analysis, with responses coded in three categories: as an overreaction (Y_i_ = 3), neither an overreaction nor an underreaction (Y_i_ = 2) or an underreaction (Y_i_ = 1). Our main exogenous measure is that of experts’ *dominant ideas,* analyzing to what degree experts support the idea of ‘focused protection’ inspired by the transnational expert discussion about the application of non-pharmaceutical interventions. Here, we relied on a question asking for agreement with the main principle of focused protection, stating that “the best approach to reaching herd immunity is to allow those who are at minimal risk of death to live their lives normally in order to build up immunity to the virus through natural infection, while better protecting those who are at highest risk”. Responses were coded in three categories including (1) strongly disagree or disagree, (2) neither agree nor disagree, and (3) agree or strongly agree. A higher value indicated a greater level of agreement with the idea of focused protection whereas a lower value indicates an inclination toward community protection. We also asked experts for their support of the alternative strategy of “community protection” by asking for agreement with the idea that “controlling community spread of COVID-19 is the best way to protect our societies and economies until safe and effective vaccines and therapeutics arrive”. Responses were coded in a similar way as to focused protection. The answers to this question correlated negatively with the focused protection question, giving us confidence that respondents saw them as alternative strategies. Focused protection and community protection maps onto the distinction between mitigation and suppression strategies. Focused protection is associated with the mitigation strategy given that herd immunity is reached by keeping R_t_ above 1 by allowing natural infection in the population while isolating individuals at risk. In contrast, a suppression strategy is associated with the strategy of community protection as non-pharmaceutical interventions like lockdowns are employed to control community spread to keep R_t_ below 1 until population-wide vaccination is possible.

We employed an ordinal logistic regression model with maximum likelihood estimates using the following equation:

$$ \ln \left(\frac{P\Big({Y}_i>j}{P\Big({Y}_i\le j}\right)={\alpha}_j+{\beta}^{\prime }X;j=1,2 $$

Where β and X are vectors of size *k*, which refer to the set of explicative variables used (see Mascia & Cicchetti 2011). The model estimates two cut-off points for *Y*_*i*_ and a single effect parameter vector (*β*_1_ 1, … *β*_*k*_) for each independent variable. The fraction on the left-side of the equation expresses the logit (i.e., probability that *Y*_*i*_ is >j versus ≤ j). There are three possibilities in Eq. (3)‘s sampling space (overreaction, neither or, underreaction).

We included four categories of control variables in the ordinal regression analysis. First, we control for politico-administrative factors relating to experts’ ‘location’ in the policy advisory system around national pandemic preparedness. We use three indicators for experts ‘closeness’ to government and ‘inside-outside’ status in public administration [14]. These are important because some policy subsystems are characterized by advisors closer to the government and the public sector having particular policy preferences, reflecting an insulated policy network [16]. The three indicators include whether experts have positions within the public health authorities or not, their seniority (do they have management/senior adviser responsibilities), and whether they have advised national governmental bodies on COVID-19 related matters during the first wave of the pandemic. We controlled for coding effects by recoding the three indicators with more fine-grained nominal categories (dummy-coded). However, this did not change the parameter estimates significantly from the one binary recode employed in the analysis.

Second, we control for epistemic factors related to associated epistemic norms or epistemic differences among experts which may be a result of similar education and training by including the disciplinary affiliation of experts in the analysis. Experts were categorized into four groups depending on their main discipline of expertise or clinical practice: (1) medicine; (2) public health, including epidemiology and biostatistics; (3) economics; and (4) what we term laboratory specializations, including molecular medicine and virology. Medicine includes doctors and other health experts engaged in clinical practice with a background in medicine whereas economics solely includes academic economists. Inspired by Jasanoff et al. [33], we distinguish between disciplines specializing in targeting social practices as in the case of epidemiology, public health, and biostatistics, and in targeting the virus on a molecular level as in the case of molecular medicine, molecular biology, and virology. It is relevant to control for epistemic factors as previous research has shown the importance of disciplinary divides for issue perception and treatment [56].

Third, we control for demographic and geographic factors such as age, gender as well as metropolitan proximity given that metropolitan areas have experienced the most COVID-19 cases. Finally, we control for sampling bias, using the previously presented measure of presence on institutional COVID-19 expert lists.

Experts’ perception of their government’s ability to save lives during the pandemic was analyzed through two different models. Model 1 tests the association of the overreaction/underreaction with country level and focused protection factors including the control variables. Model 2 includes interactions between country variables and focused protection. Analyses were performed using Stata version 16.

## Results

Denmark, Norway, and Sweden all produced pandemic preparedness plans in the early 2000s with reference to the WHOs growing emphasis on preparedness [21]. Their plans have since undergone a number of transformations, in particular, as a result of the H1N1 pandemic, which early on exposed weaknesses in health crisis management in the Nordics [3]. The COVID-19 pandemic has exposed new divergences between the Nordic countries and the importance of the politico-administrative organization of pandemic preparedness for pandemic response.

All three preparedness systems are constructed on the principles of sector responsibility (administrative units are responsible for preparedness within their purview), equality (operations should be organized similarly during emergencies as under normal conditions) and proximity (emergencies are to be handled at the lowest possible administrative level). Yet, they also differ in the responsibilities and tasks given to different layers of government and whether central authorities and political leaders can intervene in the affairs of lower levels of government and overrule the advice of public health agencies.

### Denmark

Denmark’s health care and preparedness system are organized in three governmental levels ( [60]). Denmark’s 98 municipalities are responsible for social services, including care for the elderly and disabled while hospital services are managed by the five regions. The healthcare system is decentralized in relation to service provision and public health, but the overall planning and regulation of health care is centralized to the national level [44]. Responsibility for pandemic preparedness lies within the Ministry of Health and is structured through ministerial governance. Ministerial governance refers to the arrangement that ministers in Denmark as well as Norway, across all policy areas, are able to intervene in and steer the daily operations of state agencies and overrule the advice of agency experts if they fall under their area of responsibility (Grønnegård et al. 2016). In Denmark, the Danish Health Authority (DHA) (*Sundhedsstyrelsen*) is the main guiding and coordinating organ in health emergencies [60], assisted by *Statens Serum Institut*, the expert organization responsible for epidemiological surveillance, analysis and modelling, which it reports to the DHA. Given the tradition of ministerial governance, the DHA, however, has little autonomy in steering policy if political leaders decide to intervene in its affairs. Pandemic preparedness is therefore a centralized matter in Denmark, with the central administration able to impose its policies on lower levels of government, which are not significantly involved in the policy area.

In January 2020, the DHA assessed the probability of COVID-19 entering Denmark to be very small, recommending neither screenings of individuals travelling from nor travel restrictions to known high-risk areas. As this likelihood grew throughout February, the DHA embarked on a containment strategy, adopting travel restrictions and voluntary quarantine measures for citizens arriving from risk zones. However, as hopes for containing the virus diminished due to rising infection rates, the government quickly exchanged its containment strategy with a suppression strategy to drastically restrict social practices. On the 11th of March, the same day the WHO officially categorized COVID-19 as a pandemic, the Danish Prime Minister announced that Denmark would undergo a nation-wide lockdown in order to “flatten the curve” and protect hospital capacity. The suppression strategy was supported by the invention of a precautionary principle, legitimizing the closure of kindergartens, educational institutions as well as restaurants, malls and other social gathering spots. Public gatherings were restricted to 10 persons and non-urgent medical treatment was postponed to expand hospital capacity. Interestingly, the political choice of a suppression strategy contrasted with the advice of the DHA, which favored a mitigation strategy. Nonetheless, the strategy was highly effective in reducing infection rates and hospitalizations, allowing the country to gradually open throughout the summer months. However, by late August, infection rates were on the rise again, indicating the beginning of a second wave, leading to new restrictions on social practices. By the 16th of December, the country went into its second lockdown phase.

Compared to Sweden, Denmark replaced its initial containment strategy with a suppression strategy as initial containment failed. While this strategy was highly intrusive in the lives of Danish citizens, the DHA initiated neither curfews nor severe fines for non-compliance as visible in other European countries. Overall, Denmark’s COVID-19 response to the first wave was well received in the public and among the majority of experts in the media. Critics focused on whether Denmark in fact was overreacting given the lethality of the virus and should, instead, have used a mitigation strategy. Besides what became known as the ‘Mink Scandal’, in which Denmark’s entire population of mink was exterminated due to concerns for a new dangerous mutation of COVID-19 spreading, conflicts mainly revolved around whether policy was supported by scientific evidence and if politicians should overrule the advice of experts despite the tradition of ministerial governance.

### Norway

Compared to Denmark, Norway’s health care and preparedness system is more decentralized. Although the healthcare system is also organized in three governmental levels, the tasks are distributed differently with the regions in charge of specialist care and the municipalities responsible for primary health care and social services [45, 62]. The Ministry of Health and Services has the national responsibility for pandemic preparedness in Norway, coordinating interventions alongside expert subordinate agencies like the Norwegian Directorate of Health (*Helsedirektoratet*) and the Norwegian Institute of Public Health (*Folkehelseinstituttet*). While these agencies are formally independent, ministerial governance is the structuring principle like in Denmark. As such, Norway’s public health experts and state agencies have less autonomy compared to, for instance, Sweden. Hospitals are run by four regional health enterprises with large degrees of autonomy, but ultimately owned and overseen by the Ministry of Health and Care Services. In contrast to Denmark, municipalities manage primary and long-term care as well as social services but are also responsible for pandemic preparedness. Together, this makes Norway’s health care system semi-decentralized. Since 2020, Norway has been organized in 350 municipalities, across 11 regions, giving them an increasingly important role in coordinating infectious disease management ([45]:9). During the COVID-19 pandemic, this allowed municipalities to introduce their own local restrictions on movement and face masks, sometimes in opposition to the recommendations of the central authorities. Municipal doctors (*Kommuneoverlæge*) are responsible for the local preparedness systems in terms of monitoring, documenting, and reporting rates and cases [57]. Given the municipalities role in pandemic preparedness, Norway’s preparedness system is thus more decentralized and locally attuned compared to Denmark. This local aspect also increases the amount of actors in the policy advisory system.

Despite differences in preparedness, Norway’s response was similar to Denmark. The Norwegian health authorities (NHA) also relied on a containment strategy throughout February 2020, hesitant to employ highly intrusive measures [11]. As the number of confirmed cases continued to grow, the NHA changed its initial containment strategy to a suppression strategy. On the 12th of March, the day after the lockdown of Denmark, Norway’s Prime Minister announced a similar lockdown of society, including mandatory closures of kindergartens, schools, colleges, and universities as well as significant parts of the business sector. Like Denmark, these measures were implemented to uphold a functional health care system and to “flatten the curve”. They were also the result of political pressure and intervention as the NHA advocated for the use of a mitigation strategy [11]. Nonetheless, Norway’s suppression strategy was effective in reducing infection rates and hospitalizations. Throughout April, May, and June, the country gradually re-opened, allowing 200 persons to gather in doors during the summer. During autumn, Norway re-introduced restrictions to limit the impact of a second wave by restricting alcohol servings and urging Norwegians not to invite guests home. In contrast to Denmark, the NHA has largely refrained from face mask requirements.

Norway’s COVID-19 response was thus similar to Denmark. The government changed its initial containment strategy to a suppression strategy as the numbers of COVID-19 cases rose, but never introduced curfews. However, compared to Denmark and Sweden, Norway has enforced the strongest quarantine rules, including fines for non-compliance. As for the reception, the handling of the outbreak has been described by local analysts as “consensual” and “based on pragmatic collaboration” ([11]:777), with expert bodies accepting political leadership’s decisions to diverge from their advice. One of the few tensions has been between the DHA and municipalities in the Northern part of Norway, which established local restrictions on movement due to low health care capacity. This contrasted with the recommendations of the NHA, exposing conflicts in the semi-decentralized organizations of Norway’s pandemic preparedness system. Yet, in comparison to Denmark and Sweden, Norway’s COVID-19 trajectory has been the least conflictual.

### Sweden

Like Denmark and Norway, Sweden’s health care and pandemic preparedness system is governed on three levels [36]. The central government is responsible for defining policy and legislation at the national level while the 21 regions are in charge of hospitals and healthcare. The country’s 290 municipalities are in charge of social services, including care for the elderly and disabled. In relation to pandemic preparedness, the two agencies, the Public Health Agency of Sweden (*Folkhälsomyndigheten*) (PHA) and the National Board of Health and Welfare (*Socialstyrelsen*) are the most important actors for national COVID-19 policy. Importantly, ministerial governance is prohibited in Sweden, representing a sharp contrast to Denmark and Norway. This means that ministers and politicians are prevented from intervening in the daily operations of state agencies, granting public health experts in the state a high degree of professional autonomy. Further, agencies have a limited mandate to enforce policy on regional and municipal levels unless they are supported by parliament. This makes the health system more decentralized compared to both Denmark and Norway. Since the passing of the Swedish Infectious Disease Act of 2004, regions in Sweden have established infectious disease units (*Smittskyddsläkare*), which, together with regional administrative boards (*Länstyrelser*), are responsible for pandemic preparedness within the region. In practice, this makes it difficult for the central authorities to intervene in the work of the infectious disease units. Finally, compared to Norway and Denmark, Sweden faces constitutional limits in the implementation of non-pharmaceutical interventions. This is partly related to the country’s constitution, which since 1974 has stipulated citizens’ right to free movement within Sweden and to leave its borders. As such, while the Swedish Infectious Disease Act of 2004 allows for imposing restrictions on individuals, it does not allow for general lockdown measures [41].

Compared to Denmark and Norway’, Sweden’s response to the first wave of COVID-19 has constituted an outlier because it did not enforce a lockdown. However, Sweden’s strategy has been far from as *laissez faire* as made out to be by its critics. Like its neighboring countries, Sweden initially relied on a containment strategy, encouraging people returning from high-risk regions to self-isolate [49]. But as the number of confirmed cases increased, Sweden did not exchange its containment strategy with a suppression strategy. Instead, it relied on a mitigation strategy [6], keeping its restaurants and bars open, limiting public gatherings to 50 persons while keepings its schools open for children under the age of 16 as the only country in Europe. This alternative strategy relied on two principles. First, a cornerstone of the approach was to protect vulnerable groups by banning visitors to nursing homes and urging individuals above the age of 70 to self-isolate. Second, the PHA put the Swedish citizen at the center of its strategy, appealing to its rationality and responsibility. Whereas Norway and Denmark ‘forced’ citizens to practice social distance through lockdown measures, Sweden relied on voluntary measures to flatten the curve such as hand hygiene and physical distance recommendations [36]. In doing so, the Swedish strategy took on a more expansive view of public health, framing its approach as more tenable with regard to citizens’ overall well-being, health, and fatigue rather than solely focusing on COVID-19 ([33]:94ff). In mid-December 2020, Sweden closed down non-essential public workplaces such as gyms and libraries and recommended the use of face masks on public transportation.

Sweden’s use of a mitigation rather than a suppression has stirred a great deal of domestic controversy. Compared to Denmark, and particularly Norway, the reception of Sweden’s strategy has been mixed as already mentioned, characterized by both public and expert support and criticism. One overarching tension has revolved around the high COVID-19 death toll in nursing homes and its connections to systemic shortcomings in elderly care and governmental inaction. A second tension has been the choice of a mitigation strategy. Groups of experts have, for instance, publicly criticized the PHA’s measures for being too soft, urging politicians to implement lockdown policies and adopt more non-pharmaceutical interventions similar to Denmark and Norway.

### Summary

Denmark, Norway, and Sweden responded in similar but also different ways to the first wave of the COVID-19 pandemic. Whereas Denmark and Norway favored non-pharmaceutical strategies trending towards suppression, Sweden relied on a mitigation strategy [23]. These strategies played out in different politico-administrative pandemic preparedness systems characterized by varying degree of autonomy given to health experts in the state, different centralization rates, legislative barriers, and political norms. These differences are shown in Table 2.

**Table 2:** Comparative summary of politico-administrative organization of pandemic preparedness systems

	Denmark	Norway	Sweden
**Autonomy of Health Expertise in the State**	Ministerial governance Low autonomy	Ministerial governance Low autonomy	No ministerial governance High autonomy
**Health Care System Centralization**	Centralized	Semi-decentralized	Decentralized
**Lowest administrative level of pandemic preparedness**	National	Municipal	Regional
**COVID-19 Strategy**	From containment to suppression	From containment to suppression	From containment to mitigation

**Fig. 1:**
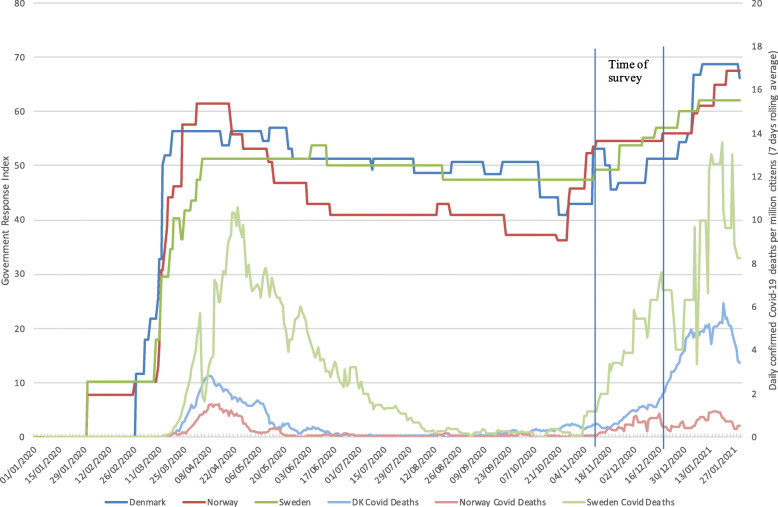
Stringency and COVID-19 Related Deaths in Denmark, Norway, and Sweden: Government containement and health response index and daily new confirmed deaths per million citizens (7 day rolling average)

Figure 1 presents a timeline of the pandemic, showing both the number of daily COVID-19 related deaths in each country as well as the strictness of their governmental response measured through the governmental response index. The index is based on eight indicators measuring the strictness and geographic scope of COVID-19 policies (e.g., school and workplace closure), and four health measure indicators (e.g., information campaigns, testing policy) normalized to a scale between 0 and 100 [29]. Figure 1 shows the difference between the suppression strategy pursued by Denmark and Norway in early March and Sweden’s mitigation strategy. During the months of May to November, the Norwegian government significantly reduced the intensity of its measures, primarily in response to low incidence rates. All countries began re-introducing stricter measures from mid-November, in response to the second wave of virus transmission. This period, however, falls outside the scope of this paper, a limitation which we will discuss later. Finally, the figure shows the timing of our survey.

### Quantitative results

Having compared Denmark, Norway, and Sweden’s pandemic preparedness systems and first wave COVID-19 responses, we turn to analyzing experts’ perceptions of their national policy responses to the COVID-19 pandemic. We start by comparing expert perception of pandemic response across countries, using our multi-dimensional measure of pandemic response. We then proceed by estimating an ordinal logistic regression model of the association of pandemic response perception on the dependent variable of “saving lives”, and expert-held dominant ideas such as focused protection, the main exogenous measure, while adjusting for potential confounding locational, disciplinary, demographic, geographic, and sampling factors.

### Evaluating pandemic response

**Fig. 2:**
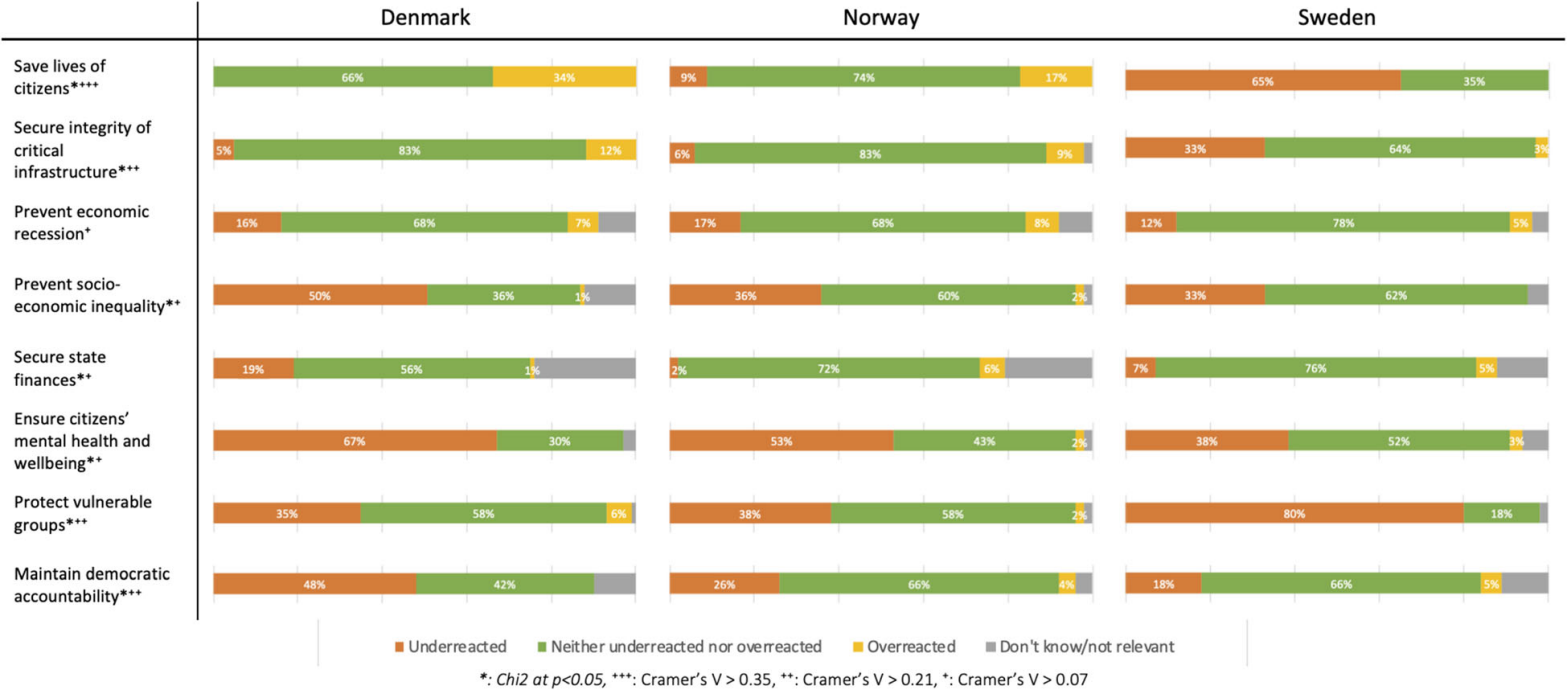
Expert Multidimensional National Response Evaluation

Figure 2 presents the results of the cross-national and multi-dimensional pandemic preparedness response measure. The measure is constructed through experts’ perception of their country’s national preparedness response (from initial detection until October 2020) in relation to eight dimensions. Comparing experts’ perceptions across the countries, similar distributions for the two dimensions of saving lives and securing critical infrastructure can be identified. However, Danish experts are more likely to perceive their country’s response as an overreaction compared to Norwegian and Swedish experts, while Swedish experts are more likely to perceive their country’s response as an underreaction on these parameters. We see a similar distribution in terms of “protecting vulnerable groups”, although very few experts perceive an overreaction on this parameter.

Danish experts are slightly more likely to perceive their government’s reaction as an underreaction on the parameter of socio-economic inequality compared to Norway and Sweden. However, all countries are primarily placed within the two categories of underreaction or appropriate reaction. Most experts perceive their government’s reaction in terms of securing state finances appropriate, but 25% of Danish experts perceive an underreaction on this parameter (that is; overspent). This matches well with the overreaction distribution reported on “saving lives”.

As Fig. 2 reveals, most experts are concerned about a governmental underreaction in terms of ensuring mental health and wellbeing of their citizens. However, Danish experts are more pronounced with 69% calling it an underreaction, vs 54 and 40% for Norway and Sweden respectively. Finally, and significantly, Norway and Sweden look very similar on the “maintaining democratic accountability” dimension, with 70% reporting an appropriate government response. However, a good half of Danish experts believe the Danish government has done too little to ensure this parameter during the pandemic.

Since the figure shows national variation along a series of dimensions, we include a measure to compare the strength of correlation between country and dimension. Cramer’s *V* is used for nominal variables that have more than two levels, and ranges from 0 to 1 [26]. The measure highlights those dimensions that show the most significant national differences. It shows the ‘saving lives’ dimension to be the most nationally divisive (with a Cramers *V* value: 0.4867, next highest is at 0.3082 for protecting vulnerable groups)*.* Of particular interest here, is the group of experts, predominantly in Denmark, but also in Norway, who consider their national pandemic response an overreaction in relation to the dimension of saving lives. Because there appear to be some confounding factors here that need explanation, we estimate an ordinal logistic regression model.

### Accounting for variation in expert perceptions

How do we explain the variation in experts’ perception of COVID-19 responses while controlling for the national responses themselves? Table 3 presents the results of an ordinal logistic regression model estimating the association between the parameter of saving lives and support for the dominant idea of focused protection, adjusting for potential confounding locational, disciplinary, demographic, geographic, and sampling, factors. Since the dependent variable has been logtransformed, effects of the explanatory variables are interpreted as changes in the log odds of perceiving pandemic response as an overreaction (versus neither underreaction nor overreaction or overreaction). The reference group for the regressions are Norwegian male experts outside of public administration who have not advised national government on COVID-19 related matters, either critical or indifferent to focused protection as a strategy, specializing in medicine, living outside of metropolises, and with seniority at the level of professor or chief physician.

**Table 3:** Ordinal regression models

	Model 1, [95% CI]	Model 2, [95% CI]
**Main exogeneous variable**		
Focused Protection	1.646*** [.9594258,2.332713]	3.035*** [.9541268,5.115762]
**Country**		
Denmark	.99** [.0745461,1.904537]	1.3** [.2262297,2.373463]
Sweden	−3.837*** [−4.982998,-2.690282]	− 3.653*** [− 4.833754,-2.472516]
**Locational factors**		
National public health institution	.328 [−.5178161,1.173866]	.379 [−.4718739,1.229217]
Seniority (management)	−.442 [−1.514791,.6311714]	−.385 [− 1.461717,.6923323]
Seniority (other)	−.701 [− 1.967297,.5651666]	−.711 [− 1.971304,.5500428]
Advised national government	.123 [−.4913383,.7370525]	.125 [−.488994,.7399688]
**Disciplinary factors**		
Discipline (ECON)	−.147 [−1.08057,.7863736]	−.127 [− 1.058946,.805931]
Discipline (LAB)	.59 [−.3414026,1.520423]	.586 [−.3430957,1.515832]
Discipline (PHEBS)	−.318 [−1.102371,.4665183]	−.346 [− 1.136617,.4455106]
**Demographic factors**		
Female	.425 [−.3013709,1.151985]	457 [−.272152,1.186631]
Age	.001 [−.0279029,.0298005]	0 [−.0286958,.0292863]
**Geography factors**		
Metropolis	−.36 [−1.035637,.3155528]	−.338 [− 1.015873,.3406098]
**Sampling factors**		
Expert list	−.348 [−1.102565,.4065451]	−.307 [− 1.062406,.448224]
**Interactions**		
Denmark*focused protection		−1.554 [−3.810335,.7018508]
Sweden*focused protection		−1.532 [−3.843457,.7792814]
/cut1	−2.923*** [−4.983256,-.8628515]	− 2.774*** [− 4.849076,-.6995892]
/cut2	2.044** [.0773675,4.010873]	2.315** [.2755515,4.353492]
Observations	232	232
*** p < .01, ** p < .05, * p < .1		

In both models, expert perception is significantly associated with support for the principle of focused protection and country of residence when controlling for locational, disciplinary, demographic, geographic and sampling factors. At standard thresholds of statistical significance, the coefficients in the models show a positive association between the principle of focused protection (e.g. disagreement with community protection) and the probability of perceiving governmental response in relation to saving lives of citizens as an overreaction. As such, experts supporting the idea of focused protection have a significantly higher probability of perceiving their government’s COVID-19 response as an overreaction compared to experts indifferent to or disagreeing with the idea. This supports previous research emphasizing the importance of dominant ideas for expert perception of pandemic response [3, 4]. However, opposed to previous studies, the results of the models do not indicate that such ideational support follows along locational nor disciplinary divides. Importantly, we find no significant covariance between support for focused protection and the locational, disciplinary, demographic, and geographic control variables. As such, the results provide little evidence of closed or contested policy networks structured along either of lines, but rather seem to show the existence of a significant minority (25%) of experts across countries, disciplines and locations in the policy advisory system strongly supporting focused protection and thereby tending to diverge from the majority support (or criticism in the case of Sweden) for national response.

The country coefficients reflect the distributions seen in Fig. 2. Compared to Norwegian experts, the reference category, Swedish experts have a significantly higher probability of perceiving their government’s efforts to save lives as an underreaction, bellying the aforementioned public expert criticism of the initial Swedish mitigation strategy. In comparison, Danish experts are more likely to perceive their government’s efforts as an overreaction compared to Norwegian experts. As reflected in Fig. 2, we find that the majority of experts in countries pursuing a suppression strategy like Denmark and Norway perceive their government’s policy response as neither an overreaction nor an underreaction while the majority of experts in a country like Sweden pursuing a suppression strategy perceive their government’s response as an underreaction. In light of the structured case comparison and the fact that only 25% of experts support focused protection, this finding highlights consistent cross-national expert policy preference for suppression strategies. While this is the general trend across the three countries, it should be noted that in both Denmark (34%) and Sweden (35%) a significant minority of experts can be identified who regard their government’s response as an overreaction (in the case of Denmark) or neither or (as in the case of Sweden). The existence of this expert minority in Denmark is most likely also the driver behind the variation between the perceptions of experts in Denmark and Norway in Table 3.

**Fig. 3:**
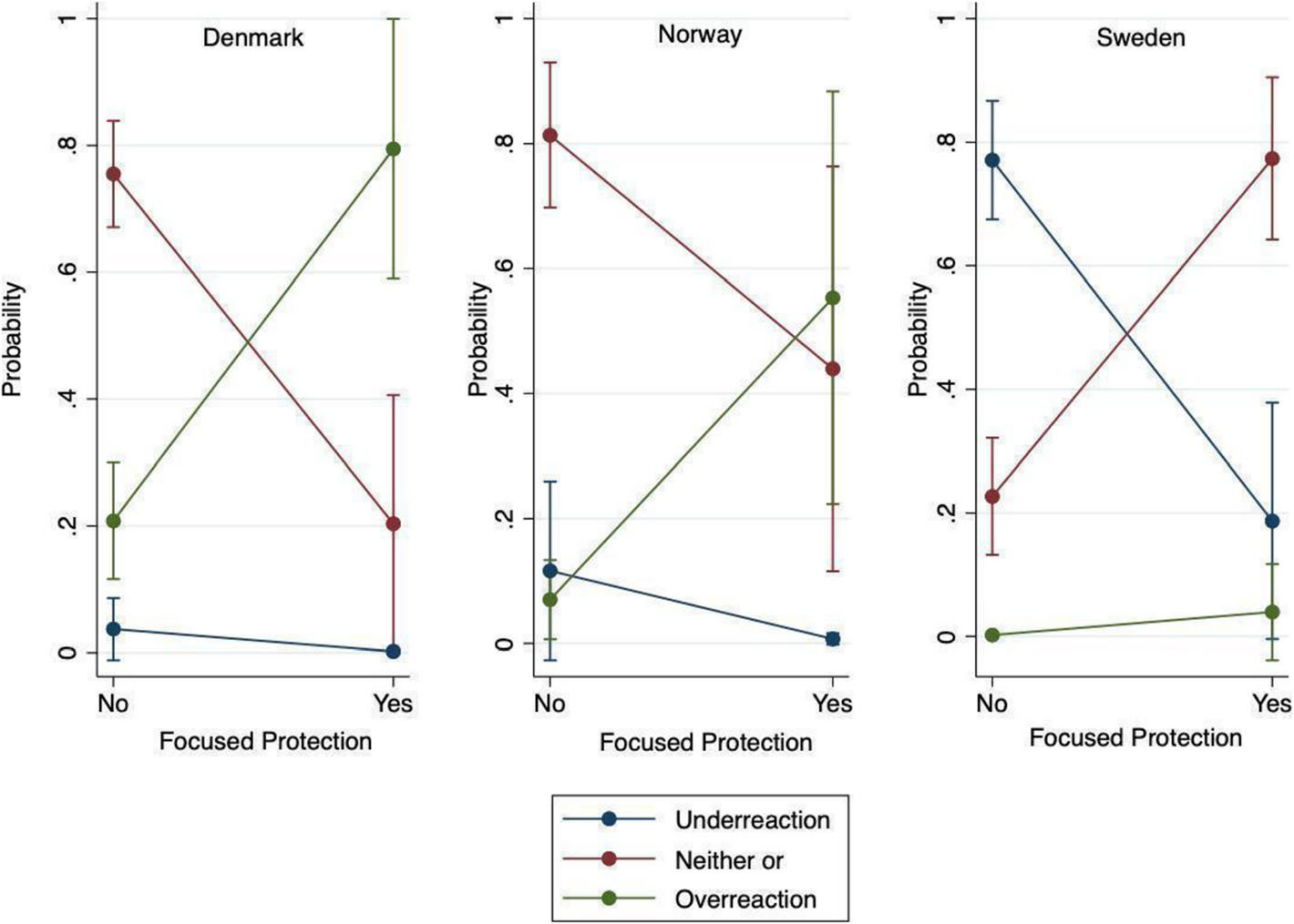
Marginal Effects of belief in focused protection as strategy on expert perception of appropriateness of government responses to save lives during the pandemic

In Model 2, we include the interactions between country variables and the focused protection variable to test whether nationality intersected with dominant ideas. Neither of these were statistically different from zero. In Fig. 3, we illustrate the effect of agreement and disagreement with the principle of focused protection on experts’ assessment of government’s ability to save lives during the pandemic, across the three countries. Figure 3 shows how experts from countries, which employed a suppression strategy during the first wave of the pandemic like Denmark and Norway have a significantly higher probability of perceiving their government’s response as an overreaction if they support the idea of focused protection. Experts from countries pursuing a mitigation strategy like Sweden are more likely to see it as neither an underreaction nor an overreaction if they support focused protection.

## Discussion

This study has, in a structured comparative way, investigated how otherwise similar countries belonging to the same public health regime like Denmark, Norway, and Sweden responded differently to the COVID-19 pandemic and to what degree their policy divergence reflect significant differences in the attitudes and policy preferences within and between national expert groups.

Our comparison suggests that the main differences between Denmark, Norway, and Sweden are not related to significant differences in the attitudes and policy preferences within and between their national expert groups. Experts present in countries pursuing a suppression strategy like Denmark and Norway systematically perceived their country’s COVID-19 response more favorably in terms of saving lives compared to experts from a country pursuing a mitigation strategy like Sweden. While showing the existence of a significant minority supporting ideas tied to a mitigation strategy, our results support findings made in other studies that point to the development of an emerging transnational expert consensus on the inadequacy of herd immunity by natural infection as a strategy [10, 58]. To take into account the politico-administrative position of actors, we control for location in the policy advisory system in the three counties through three parameters – part of national public health institution, seniority, and whether the expert has advised governmental and state institutions on COVD-19 related matters – but find that none of these significantly explain variation in how experts perceive COVID-19 policy response nor support focused protection. In relation to the literature on policy advisory system, our results show how experts’ perceptions and dominant ideas are not structured along locational lines related to either inside-outside or close-far positions in the system [14]. As such, we find no significant evidence to suggests that the policy divergence observed between Denmark, Norway, and Sweden reflect significant differences in the policy preferences of different national expert groups. Based on the first part of the results section, this leads us to suggest that other factors besides expert advice dissensus better explain COVID-19 policy divergence between countries belonging to the same public health regime such as the politico-administrative organization of national pandemic preparedness policy subsystems. One important difference identified by the structured comparison of Denmark, Norway, and Sweden’s politico-administrative organization of pandemic preparedness systems is the varying degree of autonomy of public health experts and agencies in the three countries in terms of defining and implementing policy measures to respond to the pandemic and the possibility of political leaders to overrule expert advice. This showcases the importance of politico-administrative factors for pandemic response and supports finding made in other cross-national studies of policy responses during COVID-19 such as Jasanoff et al.’s [33] comparison of 16 countries, which emphasized how policy response is conditioned by pre-existing structures in health, economic, and political systems.

Although our results indicate that experts’ perceptions of COVID-19 response and their support for policy preferences of either focused- or community protection are not structured along neither locational nor disciplinary divides, the results should be interpreted with caution as our measures of expert location in the policy advisory systems focus mainly on the structural aspects of location, thus overlooking potential other locational factors relevant for experts’ perceptions of response. One group of relational factors that the study is not able to control for are social network factors related to experts’ location in specific policy and expert networks in the policy advisory system [63]. Differences in experts’ perceptions of COVID-19 response and support for dominant ideas may be correlated with embeddedness in specific networks that cut across the locational, disciplinary, geographic, and demographic lines analyzed in the study. We suggest that the importance of social networks could be one fruitful avenue for future research to explore in order to further our understanding of the ideas and ties experts draw upon to understand health emergencies.

Further, our analysis identifies a statistically significant relationship between experts’ perception of COVID-19 policy response and dominant ideas such as the efficacy of focused protection strategies. While support for suppressive non-pharmaceutical interventions are widespread in the sample population, we identify a smaller group of experts whose support for focused protection reverses their perception of pandemic responses. Experts from countries pursuing a suppression strategy were more likely to perceive their national response as an overreaction in relation to the dimension of saving lives if they supported the idea of focused protection whereas experts from countries pursuing a mitigation strategy were more likely to perceive their response as adequate if they supported focused protection. The opposite pattern is visible for experts supporting the idea of community protection. As such, the results of the analysis highlight how support for dominant ideas like focused protection is significantly associated with how actors perceive and assess pandemic response. This finding echoes the general importance of ideas and beliefs for experts’ perception of pandemic response, which has also been highlighted by other studies [2, 3]. This study contributes to this literature by not only emphasizing the importance of ideas, beliefs, and preferences for how experts think about pandemic policy but also by adding how ideational support is correlated with other factors such as disciplinary background and location in policy advisory systems. As already mentioned, we find neither of these factors to be statistically significant. Interestingly, this contrasts with established theories of policy advice, which have identified the presence of shared norms, beliefs, and preferences in locational and disciplinary communities [32, 40]. This opens up space for hypotheses about what other factors are associated with ideational support like the aforementioned network factors.

While the study does not provide evidence of either locational nor disciplinary factors for experts’ perception of COVID-19 response nor support of either focused- or community protection, it does raise a puzzling question about the transnational diffusion and national embeddedness of dominant ideas such as focused protection, given the fact that its proponents are mainly located in Denmark and Sweden. This geographical concentration may be a result of sampling bias but it also opens up for thinking about how transnational ideas and advice about pandemic response travel and the transnational institutions that shape how experts perceive and think about pandemic response. It is beyond the scope of the present paper to provide a genealogy of both focused and community protection and their historical-institutional trajectories, but two areas for future research stand out in light of the results of the analysis. The first is related to the role and credibility of transnational expert authorities like the WHO in disseminating dominant ideas about pandemic preparedness and response. Despite recurring periods of crisis like the West Africa Ebola Outbreak in 2014–2016, the WHO has been central in shaping the transnational agenda on pandemic preparedness through the dissemination of new concepts, procedures, and vocabularies [39]. Following the publication of the Great Barrington Declaration, which strongly advocated for focused protection, the WHO’s director-general Tedros Adhanom publicly warned against the idea of herd immunity through natural infection, framing it as scientifically and ethically problematic due to the mortality rate of the disease, the possibility of multiple infections and the long-term health problems associated with infection [61]. Despite such clear indication from the WHO, a quarter of our sample supported the idea of focused protection one month after Adhanom’s statement. Combined with the high degree of policy variation observed during the COVID-19 pandemic, which occurred despite the WHO’s historical efforts to harmonize national pandemic preparedness systems, future research should focus on how experts across borders perceive the WHO and its epistemic legitimacy to better understand the sources and transmission of policy advice. The second is related to what alternative and potentially competing transnational institutions to the WHO that are able to shape experts’ support for dominant ideas like focused- or community protection. While it is not possible to compare the signature databases of the Great Barrington Declaration and the John Snow Memorandum with the results of our cross-Nordic expert survey, we identify similar contrasting policy preferences in the sample as in the two signature collections. While the majority of experts support non-pharmaceutical interventions, the existence of a significant minority of 25% experts in the survey still raises the question of how these experts came to support this idea and through what transmission channels. Here, future research could focus on exploring how these different policy preferences are related to epistemic disagreements and competing institutions within the field of epidemiology and their relationship to different political and moral values and beliefs.

Comparing how experts in similar public health regimes perceive pandemic response in situations of pandemic policy divergence can provide only partial accounts of how and why countries respond differently to pandemics because different cultural, historical, and economic policies can impact pandemic response in various ways. Future academic inquiry can extend the present study to other clusters of countries with similar public health regimes while keeping in mind that public health regimes are more fragmented and multi-level when it comes to pandemic preparedness than usually considered [38]. For example, the research design developed in this study can be extended to public health regimes outside of Europe such as East Asian welfare states [35] to compare expert perceptions of different public health regimes and interventions; this can provide inspiration for similar approaches tracing the impact of different dominant ideas on diverging pandemic responses and their support among national experts.

### Limitations

Our results should be interpreted in light of three important limitations. First, the survey was conducted in situ between November and December 2020. Although experts were asked about pandemic preparedness and response until October, it is nonetheless important to note that the expert’s perceptions may be in part a function of the timing of the study. While this is the premise of the study, that it allowed us to collect expert perceptions *in action*, thus limiting effects of retrospection, it still restricts our analysis to the first wave of COVID-19 and the information available to experts at that moment in time. This also means that the study lends itself well to future longitudinal comparisons. Second, as with most survey research of this kind, the sampling and responses are subject to potential self-selection biases. Besides controlling for sampling bias by including individuals present on pandemic expert lists, which revealed no major differences in perceptions, we have no meaningful way for controlling for such a bias. In terms of self-selection bias in survey responses, one could suspect that the survey appealed to more “critical” voices. The aim was to establish an interdisciplinary and inter-institutional sample of experts, and responses are reasonably representative in terms of gender, discipline, institutional affiliation, and seniority compared with the population (See Appendix A). Third, it is worth stressing that the survey is primarily a measure of experts’ perceptions of pandemic preparedness performance, and not an evaluation of the performance itself. Thus, we would be hesitant, and consider it premature, to conclude on the relative performance of the Nordic countries. As our survey reveals, such conclusions are difficult, not least since indicators relate to different moral choices that might be more or less valued in each polity, and the pandemic is far from over.

## Conclusion

This study has examined how countries with the same public health regime diverged in policy response to the COVID-19 pandemic and to what degree this divergence reflects important differences in the policy preferences within and between national expert groups. Drawing on pandemic preparedness plans, national health care systems, COVID-19 policies, and a cross-Nordic expert survey, three main conclusions can be drawn from the study. First, Denmark, Norway, and Sweden responded differently to the COVID-19 pandemic. In Denmark and Norway, the governments enforced suppression strategies highly dependent on non-pharmaceutical interventions like societal-wide lockdowns during the first wave of the pandemic. In Sweden, a mitigation strategy was used that focused on voluntary measures, highlighting the importance of individual responsibility and seeking herd immunity in the population while only protecting individuals at high risk. These divergences crystallized during the first wave of the pandemic despite the three countries similarity in health care and welfare state models. Second, we find little evidence to indicate that this COVID-19 policy variation between Denmark, Norway, and Sweden reflect significant differences in the policy preferences of national expert groups. When controlling for locational positions in politico-administrative systems, disciplinary affiliations, as well as demographic and sampling factors, we find that experts present in countries pursuing a suppression strategy like Denmark and Norway systematically perceived their country’s COVID-19 response more favorably in terms of saving lives compared to experts from a country pursuing a mitigation strategy like Sweden. This finding points to an emerging consensus on the inadequacy of herd immunity by natural infection as a strategy. In light of our comparison of the three countries pandemic preparedness systems and the result of our analysis of expert attitudes to pandemic responses, we suggest that policy divergence is more related to politico-administrative factors than policy preferences among national expert communities. Third, perceptions of pandemic response are associated with dominant ideas such as belief in focused protection. Experts supporting the idea of focused protection perceived their country’s COVID-19 response as an overreaction if they lived in a country pursuing a suppression strategy whereas they perceived it as adequate if they lived in a country pursuing a mitigation strategy. We find that support for a focused protection strategy is associated with a consistent policy preference across locations in the policy advisory systems, disciplinary affiliations as well as gender, age and metropolitan proximity.

The study makes a substantive contribution to existing scholarship on public health security, and the growing litterature on the COVID-19 pandemic, by comparing attitudes and policy preferences for national pandemic response among different national experts belonging to a similar public health regime. Recognition of the importance of experts in pandemic response should be part of current and future research agendas that focus on understanding variations in how global ideas of pandemic preparedness are materialized in public health regimes and politico-administrative systems. What is certain is that experts and expertise will have many and complex effects on pandemic preparedness systems and pandemic response in the future [5]. Closely documenting the effects of expert networks tasked with managing and directing systems able to respond effectively to pandemic not only enhances their accountability but can also inform future policy prescriptions.

## Supplementary Information

**Additional file 1.** Appendix A: Methods.

## Data Availability

The dataset used for analyzing the stringency and daily COVID-19 cases in Denmark, Norway, and Sweden in the current study are available in the Our World in Data database, Oxford University, https://ourworldindata.org/grapher/covid-stringency-index The other datasets used and analyzed in the study are available from the corresponding author on reasonable request.
