# Supplementary material for: Pandemic preparedness systems and diverging COVID-19 responses within similar public health regimes: a comparative study of expert perceptions of pandemic response in Denmark, Norway, and Sweden
Source: Global Health. 2022 Jan 21;18:3. doi: 10.1186/s12992-022-00799-4 (PMC8778498; doi:10.1186/s12992-022-00799-4)
Supplement: Supplementary file 1 — Additional file 1. Appendix A: Methods. [file 12992_2022_799_MOESM1_ESM.docx]

# Appendix A: Methods

## 1. Sampling of experts from expert database

This specific group of health and economic experts that received the survey were extracted from a larger cross-national database of COVID-19 experts in Denmark, Norway, and Sweden, which we constructed in summer/fall of 2020. The database contains lists of public experts on COVID-19, which we define as individuals who have a formal position at a university, hospital, research institution or public agency and offer their expert assessment of pandemic causes, effects and policies in the public domain. The database of experts was developed in three steps from public media presence:

First, “presence in the public sphere” was operationalized to presence in national newspapers. Newspapers were chosen as the primary indicator for public sphere presence as they provide a stable empirical source for comparative analysis across the countries, and have shown to be an important site for expert statements, contestation, and discussion during the COVID-19 crisis (Hede et al. 2020). We collected articles from the 8 most read nation-wide newspapers in each country, published between 01.01.2020 - 31.10.2020, which included expert statements about the pandemic. Practically, we used the media databases “Infomedia” for Denmark, and “Retriever” for Norway and Sweden. These databases collect all articles and opinion pieces published in national media and make them available for text mining and search. We searched the databases for all articles using the searchterms (‘COVID-19’ OR ‘corona/korona’) AND (‘expert’ OR ‘doctor’ OR ‘professor’ OR ‘associate professor’ OR ‘researcher’, and similar expert related keywords) in each language. In Denmark, we used the name-recognition function of the database to collect all named experts. In Norway and Sweden we downloaded all articles and used a Named-Entity recognition algorithm to elicit all names. This process yielded around 10.000 names across all countries.

Second, a team of three researchers manually refined these names in an iterative process to build an “expert database”. Names occurring more than twice were checked by at least two coders. Individuals were included if they had a formal association with a university, hospital, research institution or public agency and talked about COVID. This was done to sort out ‘self-appointed experts’ as well as private interests, and to establish a firm rule of inclusion. On top, individuals had to work in the country as we were interested solely in national expert publics. Experts from the WHO or ECDC and other international organizations were thus identified but not pursued further, although we have measures for the amount of international experts in the different national media cultures. We introduced a further inclusion rule for experts to receive the expert survey. Experts had to have a background in public health, medicine, epidemiology, virology, molecular biology, biostatistics, or economics. These professional backgrounds were selected as they matched the economic and health dimensions of preparedness, which the survey sought to address. Finally, To control for selection biases associated with public media presence, an endogenous measure in the form of “presence on institutional expert lists” was incorporated into the design. Many universities and hospitals have published lists of institutionally identified experts in light of the pandemic. We collected publicly available lists from institutions represented by a minimum of two experts in the initial dataset. The figure below represents the conceptual sample space with the dotted lines enclosing our selected sample.


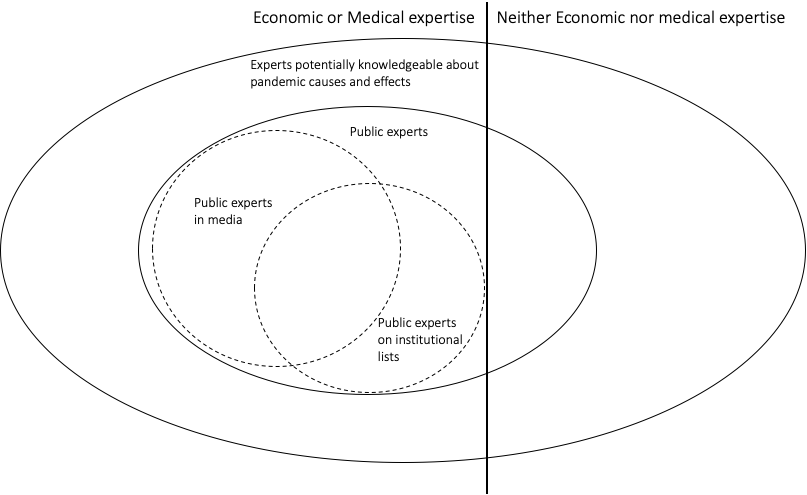


While these sampling strategies allow for a broad inclusion of different forms of expertise due to a decision rule based on institutional affiliation, ranging from health, the humanities to social science and natural science, the dataset should not be viewed as wholly representative of the countries considered. That is, it is possible that additional experts exist that we were unable to capture given the selected keywords and our focus on nation-wide media. Yet, to our knowledge, this study is the first to systematically map over time the frequency and display of different forms of expertise related to global health emergencies in the Nordics. We have researched this topic extensively, and have not yet identified any important experts that were not in our database.

Third, contact information was collected for all the sampled experts and their institutional and disciplinary affiliations coded using public profiles, CVs and job descriptions. These codes were checked three times by different coders to ensure validity, and the source of information is logged and associated with each individual. Based on this population sampling strategy, we identified 982 experts that were sent an online survey via email, open from November 17 to December 20, with two reminder mails sent to participants. The survey is available in appendix C. During analysis, we conducted a nonresponse bias analysis, to see if there were major groups of experts in the dataset that did not respond to the survey.

## 2. Nonresponse bias analysis

We conducted a nonresponse bias analysis by comparing the distributions of invited experts and survey respondents on a series of background variables; country, gender, institutional affiliation, seniority and discipline. The analysis reveals slight nonresponse bias of some groups, but very few below a 20% response rate. Notably, female experts are slightly underrepresented in our analysis. So are experts from management positions generally, and hospital positions in particular. These institutional locations have been put under the most strain during our survey period, which would explain a lack of time and interest to answer a survey.

**Table 1:** Response rates country

|  | Experts in database | Experts completed survey | Response rate per subgroup |
| --- | --- | --- | --- |
| Sweden | 440 | 91 | 21% |
| Denmark | 302 | 88 | 29% |
| Norway | 240 | 53 | 22% |

**Table 2:** Response rates gender

|  | Experts in database | Experts completed survey | Response rate per subgroup |
| --- | --- | --- | --- |
| Male | 666 | 169 | 25% |
| Female | 316 | 63 | 20% |

**Table 3:** Response rates primary institutional affiliation

|  | Experts in database | Experts completed survey | Response rate per subgroup |
| --- | --- | --- | --- |
| Universities | 623 | 160 | 26% |
| Hospitals | 156 | 30 | 19% |
| Public administration or other research | 203 | 43 | 21% |

**Table 4:** Response rates seniority

|  | Experts in database | Experts completed survey | Response rate per subgroup |
| --- | --- | --- | --- |
| Senior (Full professorship or Chief Physician) | 552 | 132 | 25% |
| Management | 121 | 24 | 18% |
| Other (less than senior or management, eg. associate/assistant prof, MD) | 339 | 79 | 23% |

**Table 5:** Response rates disciplinary affiliation

|  | Experts in database | Experts completed survey | Response rate per subgroup |
| --- | --- | --- | --- |
| MED: Infectious medicine, other branches of medicine targeted at the level of the body | 511 | 110 | 22% |
| PHEBS: Public health, Epidemiology, Biostatistics | 175 | 57 | 33% |
| ECON: Economics, Health Economics | 151 | 36 | 24% |
| LAB: Virology, Molecular biomedicine and other specialities targeting the cell level | 145 | 30 | 21% |
